# Public Health and Budget Impact of Probiotics on Common Respiratory Tract Infections: A Modelling Study

**DOI:** 10.1371/journal.pone.0122765

**Published:** 2015-04-10

**Authors:** Irene Lenoir-Wijnkoop, Laetitia Gerlier, Jean-Louis Bresson, Claude Le Pen, Gilles Berdeaux

**Affiliations:** 1 Utrecht University, Department of Pharmaceutical Sciences, Utrecht, The Netherlands; 2 Public Health & Scientific Relations, Danone Company, Paris, France; 3 IMS RWES HEOR, Vilvoorde, Belgium; 4 Université Descartes, Hôpital Necker-Enfants Malades, Paris, France; 5 Université Paris Dauphine, Paris, France; Institut Pasteur de Lille, FRANCE

## Abstract

**Objectives:**

Two recent meta-analyses by the York Health Economics Consortium (YHEC) and Cochrane demonstrated probiotic efficacy in reducing the duration and number of common respiratory tract infections (CRTI) and associated antibiotic prescriptions. A health-economic analysis was undertaken to estimate the public health and budget consequences of a generalized probiotic consumption in France.

**Methods:**

A virtual age- and gender-standardized population was generated using a Markov microsimulation model. CRTI risk factors incorporated into this model were age, active/passive smoking and living in a community setting. Incidence rates and resource utilization were based on the 2011-2012 flu season and retrieved from the French GPs Sentinelles network. Results of both meta-analyses were independently applied to the French population to estimate CRTI events, assuming a generalized probiotic use compared to no probiotics during winter months: -0.77 days/CRTI episode (YHEC scenario) or odds-ratio 0.58 for ≥1 CRTI episode (Cochrane scenario) with vs. without probiotics. Economic perspectives were National Health System (NHS), society, family. Outcomes included cost savings related to the reduced numbers of CRTI episodes, days of illness, number of antibiotic courses, sick leave days, medical and indirect costs.

**Results:**

For France, generalized probiotic use would save 2.4 million CRTI-days, 291,000 antibiotic courses and 581,000 sick leave days, based on YHEC data. Applying the Cochrane data, reductions were 6.6 million CRTI days, 473,000 antibiotic courses and 1.5 million sick days. From the NHS perspective, probiotics’ economic impact was about €14.6 million saved according to YHEC and €37.7 million according to Cochrane. Higher savings were observed in children, active smokers and people with more frequent human contacts.

**Conclusions:**

Public health and budget impact of probiotics are substantial, whether they reduce CRTI episodes frequency or duration. Noteworthy, the 2011-12 winter CRTI incidence was low and this analysis focused on the fraction of CRTI patients consulting a practitioner.

## Introduction

Common respiratory tract infections (CRTI) include common cold (CC), upper respiratory tract infections, influenza like illness (ILI) and flu [1–3]. CRTI are mainly of viral origin, are contagious and transmitted via airborne droplets, direct contact or through contaminated objects [4–5]. Symptoms include runny nose, sneezing, sore throat, coughing, and sometimes fever, most of the time self-limited and usually resolving in seven to ten days. On average, adults have two to five infections annually and children typically present six to twelve “colds” per year [1, 6]. Rates of symptomatic infections increase in the elderly. Overlapping clinical presentations among influenza, CC, upper respiratory tract infections and flu make differential diagnosis difficult [7–8].

Over-the-counter medications can bring relief of symptoms [9], but do not alter the course of the disease. Antibiotics are recommended only in the case of superinfection [10].

Although the average cost of a CRTI episode is low, the high incidence and the recurrence rates lead to a high burden for the national health systems (NHS), CC being the most common reason for visiting general practitioners (GP) and for antibiotic prescription in children. In addition, CRTI recurrences affect parents’ quality of life [11]. Lastly, non-medical direct costs (e.g. need for babysitting) and absenteeism represent a significant burden [12, 13].

Nutritional intervention trials have investigated the benefits of many different probiotics in the management of CRTI [14–24]. The growing number of studies assessing the impact of probiotics-based interventions reflects the need for a proper measurement of the probiotics effects on given clinical symptoms and/or disease burden. The recent constitution of dedicated interest groups within the Health Technology Assessment international (HTAi) organization and more recently by the International Society for Pharmacoeconomics and Outcomes Research (ISPOR) confirms the key role of nutrition, including probiotics, among the possible Public Health strategies [25, 26]. Probiotics are easily available for the general population through daily consumption of fermented dairy products or food supplements. At the beginning of this century, the Food and Agriculture Organization (FAO) and World Health Organization (WHO) defined probiotics as “Live microorganisms which when administered in adequate amounts confer a health benefit on the host” [27]. This definition was confirmed recently by an expert consensus group [28]. The likely mechanism of probiotic impact on CRTI is through bolstering immune response; several studies have shown probiotics to increase the numbers of T-lymphocytes and to enhance phagocytosis, natural killer cell activity, and IgA production [29]. Probiotic health effects are often regarded as strain-specific. However the results of many meta-analyses, including the two studies applied in this paper, pool data on similar clinical outcomes achieved by different *Lactobacillus* and *Bifidobacterium* species and strains. This supports the concept that some effects may be common among a range of strains. Recently, experts supported this concept for several public health benefits associated with a cross section of probiotics [30].

Three meta-analyses were conducted in the area of CRTI. The first one, from the Cochrane Library, stated that “… probiotics were better than placebo when measuring the number of participants experiencing episodes of acute upper respiratory tract infection” [31]. Comparable results were reported by the York Health Economics Consortium (YHEC) who conducted a systematic review followed by a meta-analysis. They found that individuals who received probiotics had significantly shorter episodes of CRTI by almost one day compared to those receiving a placebo [32]. The third one focused on the prevention of common colds and excluded other upper respiratory tract diseases. It showed a protective effect of probiotics of borderline significance [33].

We hypothesize that reducing the duration or the incidence of CRTI during the winter season will influence health care utilization and associated expenditures in Western Europe countries.

## Objectives

The purpose of our study was to estimate the public health impact and related budget consequences of probiotics use, pertaining to a reduction of the duration (YHEC scenario) or the incidence (Cochrane scenario) of CRTI, applied to France as a representative Western Europe country.

## Methods

This analysis was conducted according to the French recommendation on methods for health economics evaluation published by the Haute Autorité de la Santé, which advises the use of models to estimate the Public Health and economic effects of new health care intervention [34]. No Ethics Committee submission nor informed consents were required for our study since it did not involve any patient recruitment nor individual records consultation. All inputs were retrieved from publicly available sources as described in the next sections.

### Model description

A state-transition microsimulation (individual-based) Markov model was developed using TreeAge Pro 2009.

**Fig 1:**
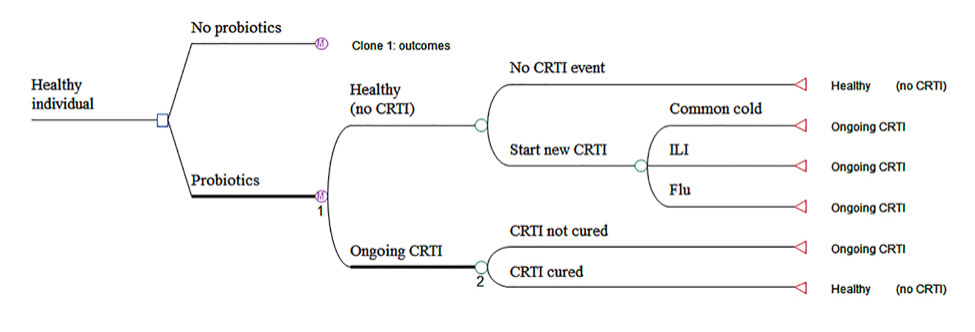
Markov model structure ‘probiotics vs. no probiotics’ (TreeAge software display). M nodes: indicates Markov nodes (starting point of simulation). Circles: indicates a chance node (probability needed). Triangle: indicates a terminal node, Square: decision node. ILI: influenza-like illness. The model compares a strategy without probiotics to a strategy with probiotics intake. All individuals were supposed healthy at model entry. Over the model course, the possible outcomes, with strategy-specific probabilities, are to develop a new CRTI or to remain healthy. In case of a new CRTI event, the cases are split into common cold, non-flu ILI, and flu. In case of ongoing CRTI, the possible outcomes are to be cured or to remain sick with CRTI.

Two health states were considered: “Healthy” and “CRTI” (Fig 1). The Markov cycle length (the time periodicity of updating all model parameters) was 1 day and time horizon was 217 days, covering a winter season from October until April to match the usual monitoring activity of the flu cases in European networks, including the French ‘Sentinelles’ [35]. A virtual population approach based on first-order Monte-Carlo simulations was used, as recommended by Gueyffier et al [36]. Healthy individuals entered the model at the beginning of the season of “winter colds”.

**Fig 2:**
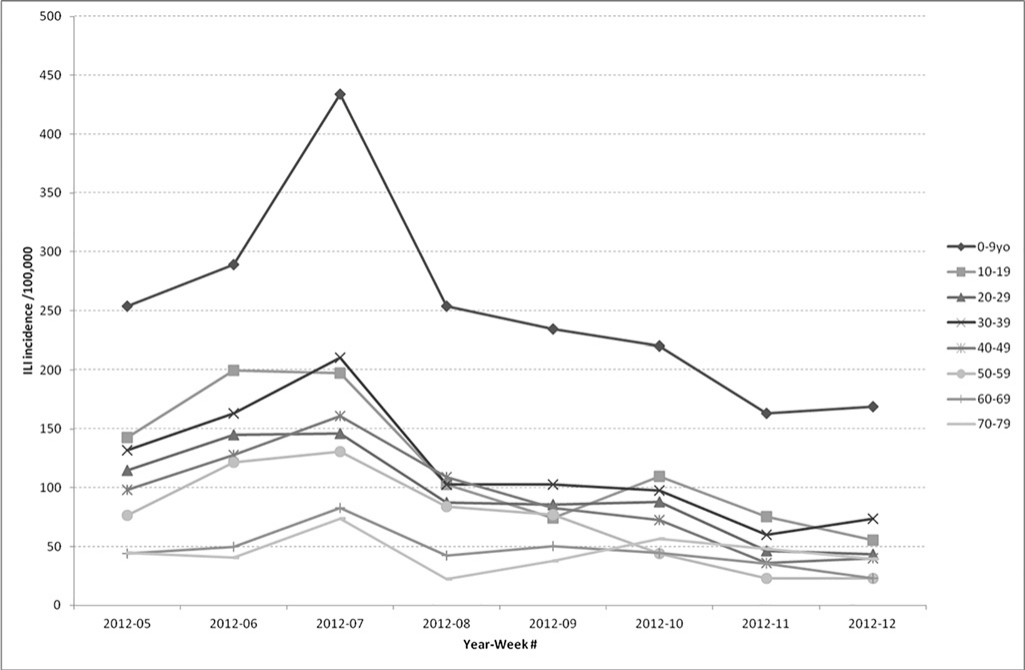
Weekly influenza-like illness incidence rate (/100,000) according to Sentinelles network by age for the epidemic period of winter 2011–2012. yo: year-old. Source: réseau Sentinelles, INSERM/UPMC, http://www.sentiweb.fr Accessed 24 March 2015. Age is a risk factor for CRTI. The figure shows that the ILI incidence during the epidemic season (weeks 5 to 12 of 2012, during winter 2011–2012) is age-dependent. Children aged between 0 and 9 years have the highest incidence rates, up to 434 cases/100,000 at season’s peak.

A sampling rate of 1/1000 represented the French population structure (by gender and 5-year age ranges to reproduce the Sentinelles data) and Sentinelles’ ILI incidence rates per age (Fig 2).

This model included neither herd immunization (probiotic effect was limited to the consumer) nor self-immunization. Therefore, cured subjects joined back the general population and its exposure to CRTI.

### Study population and comparator

The population was representative of the French population, according to demographics and known risk factors of developing CRTI (age, passive or active smoking, living in a community setting). The evaluated strategy was a generalized (100%) use of probiotics among the French population aged 3 to 79 years-old while the reference strategy was placebo i.e. the absence of probiotics use. The maximal probiotic effect was therefore estimated, in the absence of published data on the current probiotics consumption.

### Epidemiological sources

The size of the French population per age group and gender in 2012 was obtained from Eurostat [37]. In 2012, the French metropolitan population was 65.4 million, of which 59.3 million aged between 3 and 79.

The Sentinelles GP network records ILI in the general French population to identify flu outbreak at a national level. ILI is defined as sudden fever higher than 39°C (102°F), myalgia and respiratory signs. It includes ‘real flu’ and ‘non flu’ ILI cases. For our broader CRTI modelling purpose, ‘non flu’ ILI and flu cases were split, and then CC occurrence was extrapolated using a large observational study which reported relative ratio of CC/flu over several winter seasons [38].

### CRTI probabilities in the general population

Three types of CRTI were considered in the model: CC, ILI and flu. The daily probability to develop a CRTI was derived from the weekly number of ILI cases reported by Sentinelles. The probability was adjusted for environmental known risk factors: age, passive or active smoking, living in a community setting.

### CRTI risk factor: age

Children and elderly were more prone to visit their doctor for ILI, as shown in Fig 2. According to Fleming and Ayres, the number of CC episodes per 1 ILI was higher in the younger age groups: 3.04 in the 0–4 year-old population, 1.73 in the 5–14, 1.05 in the 15–44, 1.09 in the 45–64 and 1.92 among those aged 65 or above [34].

### CRTI risk factor: smoking status

Bensenor et al. found a significant increase in the average duration of a CC episode in active smokers (Relative Risk [RR] of duration >7 days = 1.62 [1.40;1.87] in light smokers, 2.63 [2.02;3.44] in smokers of ≥25 cigarettes per day) [39]. CRTI duration was assumed to be +16.8% in active smokers. In passive smokers, a significantly longer duration of CRTI (adjusted RR of having a cold lasting over 7 days = 1.12 [0.99;1.27] vs. never smokers) and a significantly higher incidence of colds (adjusted RR of having at least one cold = 1.15 [1.05;1.26] vs. never smokers) were reported. CRTI duration was assumed to be +4.5% in passive smokers vs. non-smokers. Prevalence of active smoking by age and gender was obtained from the French National Institute for Statistics and Economic Studies (INSEE) in 2010 [40]. The probability to be a passive smoker among the non-smoking population was fixed as equal to the proportion of active smokers in the general population (Table 1).

**Table 1:** Summary of model inputs—Epidemiological parameters.

Model parameters	Value	Sampling information	Reference
Season start-end	Oct 2011-Apr 2012		Sentinelles definition
Time horizon (days)	217		Season duration
French population size 3–79 yo	59,316,541	Rate 1/1000	Eurostat 2012
**Risk factors**	**% population**	**Sampling information**	**Reference**
* **Age (years)**		uniform, /sex, age group	
3 to 9	9.6%		Eurostat 2012
10 to 24	20.3%		
25 to 64	57.3%		
65 to 79	12.8%		
* **Active smoker**	24.5%	uniform, /sex, age group	OFDT 2010
* **Passive smoker**	18.5%	uniform, /sex, age group	Assumption
* **Living in a community setting**		uniform, /sex, age group	
Attending school (3–9)	99.5%		DEPP 2010–11
Students (10–24)	79.1%		DEPP 2010–11
Employed, in open-space (25–64)	35.8%		INSEE 2011
Living in an institution (65–79)	2.8%		EHPA 2003
**RTI characteristics**	**Incidence/100,000**	**Duration (days)**	**Reference**
CC	2,429	7	Fleming & Ayres 1988 (N CCs:ILI)
ILI	1,758	7	Sentinelles CRTIs—flu cases
Influenza	1,548	7	Fleming & Ayres 1988 (N flu:ILI+flu)
**Total CRTI incidence**	5,735	7	Sentinelles 2011–12 + Fleming & Ayres 1988
**Impact of risk factors:**	***on CRTI incidence***	***on CRTI duration***	**Reference**
* **Age (years)**	*per 100*,*000*	*N days*	
3 to 9	13,347	7	Sentinelles 2011–12; YHEC 2012
10 to 24	5,960	7	Sentinelles 2011–12; YHEC 2012
25 to 64	4,975	7	Sentinelles 2011–12; YHEC 2012
65 to 79	3,098	7	Sentinelles 2011–12; YHEC 2012
* **Active smoker**	NA	+4.5% vs. no smokers	Bensenor 2001
* **Passive smoker**	RR = 1.15 vs. no smokers	+16.8% vs. no smokers	Bensenor 2001
* **Living in a community setting**			
Day care (e.g. school) vs. home care	RR = 1.22	NA	Louhiala 1995
Shared office vs. alone	RR = 1.07*	NA	Jaakkola 1995
**Impact of using probiotics:**	***on CRTI incidence***	***on CRTI duration***	***on AB use***
Reference YHEC 2012	NA	-0.77 days vs. pbo	NA
Reference Hao 2011	RR = 0.72 vs. pbo**	NA	RR = 0.67 vs. pbo

EHPA: Elderly hosting institutions; DEPP: Directorate for assessment and forecasting and performance; INSEE: National Institute of Statistics and Economic Studies; OFDT: French Monitoring Centre for Drugs and Drug Addiction; SPILF: Society of Infectious Pathology of French language; yo: year-old

*OR = 1.64;

**OR = 0.58.

Conversions into RR using exact numbers of events and sample sizes.

### CRTI risk factor: living in a community setting

Children between 1 and 7 years old attending day care centers had significantly more CC (RR = 1.22 [1.13;1.31]) compared to children in private home care [41]. Adults working in shared office had an increased risk of developing CC compared to those working in individual offices (adjusted OR for having 2 or more CC = 1.35 [1;1.82]) [42]. The OR for having at least one CC in the past 12 months was 1.64 [1.08;2.49]. The rates of school and university attendance and of adults working in a shared office were retrieved from INSEE [40].

### Effect of probiotics on CRTI

Two simulations were conducted independently, using the results from the two meta-analyses. Both analyses are thus based on different assumptions.

#### YHEC scenario

In a first scenario, the results from the YHEC meta-analysis were used. Based on 9 studies (see main characteristics in Table 2), including a total of 1577 probiotics-receiving vs. 1774 placebo-receiving individuals, the mean duration of CRTI illness was 0.77 days [-1.50;-0.04] shorter in the probiotic arm (p = 0.04). The average duration of a CRTI episode was 7 days in the placebo group, which was the duration used in our “no probiotics” control arm [32].

**Table 2:** Main characteristics of the studies included in the YHEC and Cochrane meta-analyses.

Reference YHEC	Country	Population	Duration	Total probiotic dose per day	Comparator
Bentley 2008 (unpublished)	Germany	Adults at increased risk of infection (at least 2 episodes in the previous 6 months)	12 weeks	1x10^9^ CFU	Placebo: sachet containing maltodextrin without living cultures.
Berggren, Lazou Ahren et al. 2011	Sweden	Healthy adults aged 18–65 years.	12 weeks	1x10^9^ CFU	Placebo: 1.0g maltodextrin powder sachet.
Cáceres et al. 2010	Chile	Children (1 to 5 years of age) attending day care centres.	3 months	1x10^8^ CFU	Placebo: milk product with no probiotic.
de Vrese. 2005	Germany	Healthy adults (aged 18–67).	3 months then 5.5 months	5x10^7^ CFU	Placebo: vitamin mineral preparation without probiotic.
Guillemard et al. 2010	Germany	Adults aged 18–65 years; working in 2- or 3-shift work patterns (including night work).	3 months	1.1 x 10^9^ CFU	Placebo: a non-fermented, acidified, sweetened, flavoured dairy drink without the active components.
Guillemard et al. 2010	France	Male and female individuals of at least 70 years of age who were free-living (not residing in an institution).	3 months	1.1 x 10^9^ CFU	Placebo: a non-fermented, acidified, sweetened, flavoured dairy drink without the active components.
Kloster Smerud 2008	Norway	Children (12–36 months) attending day care centres.	7 months	1.5 x 10^10^ CFU	Placebo: ordinary fermented milk drink heated to 75 degrees Celsius for 4 seconds to ensure absence of probiotic bacteria (raspberry flavoured).
Niborski et al. (unpublished)	France	Healthy adults (mostly men).	7 weeks	NA	Placebo: acidified milk (no bacteria).
Turchet et al. 2003	Italy	Free-living elderly people over 60 years of age.	3 weeks	1x10^9^ CFU	No study product.
**Reference Cochrane**	**Country**	**Population**	**Duration**	**Total probiotics dose per day**	**Comparator**
Berggren 2010	Sweden	Health volunteers aged 18 to 65	12 weeks	1× 9 10~9 CFU	Placebo
Hojsak 2010a	Croatia	Children aged 13 to 86 months attending daycare centre.	4 months	10~9 CFU	Same post-pasteurised fermented milk product
Hojsak 2010b	Croatia	all patients older than 12 months and hospitalised at the paediatric department	Hospitalisation duration (average 5 days)	10~9 CFU	Same post-pasteurised fermented milk product
Kekkonen 2007	Finland	those who participated in the Helsinki city marathon.	3 months	4 × 10^10^ bacteria (bottle) Or 1.0 × 10^10^ CFU (capsules)	Placebo
Rautava 2009	Finland	0 to 2 months infants	12 months	1 × 10^10^ CFU	Placebo
Sanz 2006	Spain	All children aged 3 to 12 studying in selected schools	20 weeks	NA	Placebo

**CFU:** colony-forming units

#### COCHRANE scenario

In a second scenario, results from the Cochrane meta-analysis were considered. The odds ratio for having at least 1 CRTI episode was 0.58 [0.36;0.92] (p = 0.022), based on 6 studies (Table 2) including a total of 940 individuals in the probiotics arm vs. 896 in the control arm. This ratio was converted into a relative risk of 0.72, and applied to the baseline CRTI risk to obtain the probability of having a CRTI in the probiotics arm of the model [31]. Note that only the likelihood of decreased symptoms was reported, and not the likelihood for the CRTI infection to be transmitted (static model).

In both meta-analyses, the included studies lasted generally for 3 months, including the winter months. The probiotic dosage was between 10^8^ and 10^10^ colony forming units (CFU) per day, via oral consumption.

### Resources utilization

The resources utilization included in the model were GP visits, antibiotics, non-antibiotic drugs prescribed and sick leave days. Mosnier et al. reported an ILI antibiotic prescription rate of 15% in children aged 1–12 and 34.1% in adults [43]. According to Hao, patients consuming probiotics had lower antibiotic prescription vs. controls (risk ratio of 0.67 [0.45;0.98] (p = 0.04)) [31].

Cohen et al. reported the number of visits and drug prescription costs in ILI patients, according to disease severity [44]. The authors defined “Mild” as the need for 1 GP consultation, no sick leave and no complication (56%), “Moderate” included a single GP visit at the patient’s home and/or a sick leave (28%), and “Severe” cases were complicated cases requiring more than 1 GP visit (16%). A weighted average cost was calculated based on the relative frequency of severity.

EcoGrippe reported that 70% of employed individuals consulting for CRTI received a sick leave prescription for an average duration of 4.8 days, and 25% of employees took a sick leave because of sick children for an average duration of 3.0 days [13].

Table 3 describes the resource use parameters included in the model and their sources.

**Table 3:** Summary of model inputs—Resource utilization parameters.

Population age ranges	3–14 yo	15–64 yo	65–79 yo	Reference
GP visits (common cold; ILI/flu)	1.1;1.4	1.0;1.2	1.0;1.3	Cohen 2001
% with antibiotics course	15.0%	34.1%	34.1%	Mosnier 2002
N distinct medications prescribed	3.7	3.7	3.7	SPILF 2005
**Unit costs (€)**	**Society**	**NHS**	**Family**	**Reference**
GP visit	31.2	15.1	16.1	ameli.fr 2013
Antibiotic course	5.2	2.9	2.3	BdM_IT 2013
Non-antibiotic drugs (range)	2.6–7.4	1.2–4.3	1.4–3.1	Cohen 2001
**Total cost per CRTI episode (€)**	**Society**	**NHS**	**Family**	**Reference**
3–14 year-old (common cold;ILI/flu)	46.7;55.6	22.4;27.0	24.3;28.7	Resource use x unit cost
15–64 year-old (common cold;ILI/flu)	45.5;52.0	22.3;25.6	23.1;26.4	Resource use x unit cost
65–79 year-old (common cold;ILI/flu)	61.4;61.9	32.7;31.8	28.7;30.1	Resource use x unit cost
Total population (common cold;ILI/flu)	47.7;53.9	23.7;26.6	24.0;27.2	Resource use x unit cost
**Indirect cost parameters**	**15–24 yo**	**25–49 yo**	**50–64 yo**	**Reference**
% employed adults	29.9%	81.6%	54.8%	INSEE 2011
**Sick leave prescriptions:**	**%**	**Mean duration (days)**	**Assumption**	**Reference**
for sick children (aged 3–14)	25.0%	3.0	Assuming parents aged 25–49 year-old	Cohen 2007
for employed adults (aged 15–64)	70.0%	4.8	See employment rates above	Cohen 2007
**Unit cost of a working day lost (€)**	**Society**	**NHS**	**Family**	**Reference**
Day loss, up to 3 days	142.5	0.0	109.6	GDP/capita, avg net income 2012
Day loss, as from Day 4	142.5	31.4	0.0	Avg daily allowance 2012

### Economic perspective

Three economic perspectives were considered: the NHS (direct medical costs paid by the public insurer), the society (NHS + indirect costs due to productivity losses) and the family (medical co-payments and revenue losses in case of sick leave). The unit costs of medications and medical visits were retrieved from the French NHS website [45]. Medical costs were updated for 2012, using the evolution of the harmonized consumer price index for pharmaceuticals between 2001 (96.14) and 2012 (101.81) [40].

An allowance of 31.37€/day was applied for sick leave according to the NHS perspective (French Court of Auditors report [46]). The French NHS does not provide daily allowance for the first 3 days of sick leave, the loss from the family perspective was estimated at 109.55€/day (annual net income in France in 2011 was 23,882€per person, for an average of 218 working days) [40]. From the society perspective, the value of a working day loss was estimated at 142.5€, obtained by dividing the gross domestic product (GDP) per capita by the average number of working days per year [40].

The cost of the probiotics from the family perspective was addressed separately and estimated using the average probiotic dosages and durations of the studies as presented by the YHEC and Cochrane meta-analyses (Table 1), combined with the cost price of probiotic products, derived from national supermarkets. Based on this, a range of cost for a 4-person family was estimated.

The public health outcomes of our model show the number of CRTI episodes (separately for CC, ILI and flu), number of CRTI-days, number of antibiotic courses, number of sick leave days. The economic outcomes included the direct medical costs (medical honoraria, antibiotics and prescribed non-antibiotics drugs) and indirect costs (productivity loss). All outcomes were estimated for the French general population likely to consume probiotics (3–79 years of age, N = 59,300,000) and are presented separately depending on the related scenario (YHEC or Cochrane).

## Results

Anchoring of the model was demonstrated; the demographics and Sentinelles data were reproduced with an error rate inferior to 5%.

The first scenario analysis (YHEC) showed that the reduced duration of CRTI by almost 1 day due to probiotics led to 2.383 million fewer days with CRTI, while 291,000 antibiotics courses were avoided and the number of prescribed sick leave days diminished by 581,000 days in the arm using probiotics compared to no probiotic consumption.

In the second scenario (Cochrane), the reduction of incidence of CRTI episodes with probiotics vs. without probiotics showed a higher overall impact on public health than a reduction of duration. Extrapolated to the French situation, the results represented a reduction of 6.639 million CRTI days, the number of antibiotic prescriptions was reduced by 473,000 and 1,453,000 prescribed sick leave days were avoided during the 2011–2012 winter season. (Table 4).

**Table 4:** Public Health impact of probiotics (model population 3–79 year-old, N = 59,300).

YHEC meta-analysis			
	Probiotics	No probiotics	Difference
N episodes CC	1,277	1,277	0
N episodes ILI	941	941	0
N episodes flu	880	880	0
**N CRTI episodes (any)**	**3,098**	**3,098**	**0**
N days CC	8,248	9,230	-982
N days ILI	6,098	6,822	-724
N days flu	5,730	6,407	-678
**N CRTI days (any)**	**20,076**	**22,459**	**-2,383**
N courses antibiotics	590	881	-291
N sick days	4,278	4,860	-581
**Cochrane meta-analysis**			
	**Probiotics**	**No probiotics**	**Difference**
N episodes CC	929	1,291	-362
N episodes ILI	683	986	-303
N episodes flu	585	838	-253
**N CRTI episodes (any)**	**2,197**	**3,115**	**-918**
N days CC	6,695	9,303	-2,607
N days ILI	4,964	7,151	-2,187
N days flu	4,260	6,105	-1,846
**N CRTI days (any)**	**15,919**	**22,559**	**-6,639**
N courses antibiotics	426	899	-473
N sick days	3,509	4,962	-1,453

To extrapolate to the France level, a factor x1000 can be applied to the above figures.

CC: common cold; CRTI: common respiratory tract infection; ILI: influenza-like illness

Savings associated with the effect of probiotic use as reported by the YHEC study were estimated at €84.4 million, €14.6 million and €16.2 million, for the society, the NHS and the family, respectively. Using the Cochrane meta-analysis results, these figures amounted to €253.6 million, €37.7 million and €131.1 million, respectively (Table 5). For the society and the family perspectives, savings were mainly generated by fewer days of sick leave. Assuming a daily consumption of one serving (100mg) of probiotics, during a period of 7 months, the average cost of probiotic consumption through currently available products was ranging from 126€ to 336€ for a 4-member family. From the NHS perspective, savings were mainly generated by fewer visits and non-antibiotic drugs, followed by fewer antibiotic prescriptions, as the cost of probiotics does not incur to the NHS.

**Table 5:** Probiotic savings according to the perspective and the meta-analyses (€ 2012), population aged 3–79 (N = 59.3 million).

Society—YHEC			
	Probiotics	No probiotics	Difference
Cost visits	148,331	148,331	0
Cost antibiotics	3,093	4,617	-1,524
Cost sick days	609,541	692,372	-82,831
**Total cost**	**760,965**	**845,320**	**-84,355**
**NHS—YHEC**			
Cost visits	72,356	72,356	0
Cost antibiotics	1,718	2,564	-846
Cost sick days	35,454	49,255	-13,801
**Total cost**	**109,528**	**124,175**	**-14,647**
**Family—YHEC**			
Cost visits	75,962	75,962	0
Cost antibiotics	1,381	2,062	-681
Cost sick days	344,720	360,194	-15,474
**Total cost**	**422,063**	**438,218**	**-16,155**
**Society—Cochrane**			
Cost visits	104,968	149,110	-44,142
Cost antibiotics	2,231	4,708	-2,477
Cost sick days	499,969	706,947	-206,978
**Total cost**	**607,168**	**860,765**	**-253,597**
**NHS—Cochrane**			
Cost visits	51,247	72,800	-21,553
Cost antibiotics	1,239	2,615	-1,376
Cost sick days	36,128	50,934	-14,806
**Total cost**	**88,614**	**126,349**	**-37,735**
**Family—Cochrane**			
Cost visits	53,711	76,296	-22,585
Cost antibiotics	996	2,103	-1,107
Cost sick days	258,143	365,534	-107,391
**Total cost**	**312,850**	**443,933**	**-131,083**

YHEC: York health economic consortium. Estimated range of average cost of probiotics: 126€ to 336€, for a 4-member family, assuming a daily consumption of one serving of 100mg during a period of 7 months.

Table 6 reports the above results according to sub-populations exposed to environmental risk factors. From a society perspective and based on the YHEC study, children aged 3–9 would benefit most from probiotics. They represent 9.6% of the population, but 20.4% of the total CRTI days saved and 14.3% of the potential health-economic savings generated by probiotics. They are followed by subjects living or working in a community setting who represent 45.3% of the population, but 52.6% of the CRTI days saved and 48.9% of the savings. Passive smokers also generated higher savings than the general population, followed by active smokers. Cochrane-based results were similar: the consumption of probiotics has a larger impact in people living/working in a community setting followed by children of 3–9 years old.

**Table 6:** Analysis by risk factors (age, smoking, living in the community), population aged 3–79, society perspective.

YHEC—Age 3–9 (9.6%)			
	Probiotics	No probiotics	Difference (% of total)
Total CRTI days	3,973	4,458	-485 (20.4%)
Cost honoraria	30,870	30,870	0
Cost AB	332	495	-163 (10.7%)
Cost sick days	43,085	54,950	-11,865 (14.3%)
**Total cost**	**74,286**	**86,315**	**-12,029 (14.3%)**
**YHEC—Passive smoker (18.3%)**			
Total CRTI days	4,370	4,888	-517 (21.7%)
Cost honoraria	32,423	32,423	0
Cost AB	631	941	-311 (20.4%)
Cost sick days	117,681	134,288	-16,608 (20.0%)
**Total cost**	**150,734**	**167,652**	**-16,918 (20.1%)**
**YHEC—Active smoker (24.5%)**			
Total CRTI days	4,867	5,393	-527 (22.1%)
Cost honoraria	31,732	31,732	0
Cost AB	820	1,224	-404 (26.5%)
Cost sick days	188,885	211,776	-22,891 (27.6%)
**Total cost**	**221,437**	**244,732**	-23,295 (27.6%)
**YHEC—Community (45.3%)**			
Total CRTI days	10,482	11,734	-1,253 (52.6%)
Cost honoraria	77,838	77,838	0
Cost AB	1,338	1,997	-659 (43.3%)
Cost sick days	248,063	288,645	-40,583 (49%)
**Total cost**	**327,239**	**368,481**	**-41,242 (48.9%)**
**Cochrane—Age 3–9 (9.6%)**			
Total CRTI days	2,898	4,097	-1,199 (18.1%)
Cost honoraria	20,075	28,417	-8,342 (18.9%)
Cost AB	216	457	-240 (9.7%)
Cost sick days	35,848	50,676	-14,828 (7.2%)
**Total cost**	**56,140**	**79,550**	**-23,410 (9.2%)**
**Cochrane Passive smoker (18.3%)**			
Total CRTI days	3,301	4,725	-1,424 (21.4%)
Cost honoraria	21,857	31,270	-9,413 (21.3%)
Cost AB	436	941	-505 (20.4%)
Cost sick days	96,022	140,385	-44,363 (21.4%)
**Total cost**	**118,315**	**172,596**	**-54,281 (21.4%)**
**Cochrane Active smoker (24.5%)**			
Total CRTI days	3,894	5,420	-1,526 (23%)
Cost honoraria	22,960	32,083	-9,123 (20.7%)
Cost AB	591	1,231	-640 (25.8%)
Cost sick days	150,517	211,592	-61,076 (29.5%)
**Total cost**	**174,068**	**244,907**	**-70,839 (27.9%)**
**Cochrane—Community (45.3%)**			
Total CRTI days	8,490	12,075	-3,585 (54%)
Cost honoraria	56,050	79,888	-23,838 (54%)
Cost AB	1,015	2,145	-1,130 (45.6%)
Cost sick days	226,803	319,391	-92,588 (44.7%)
**Total cost**	**283,868**	**401,423**	**-117,555 (46.4%)**

RTI: respiratory tract infection; YHEC: York health economic consortium.

## Discussion

A model was developed to extrapolate the results of two meta-analyses investigating the effect of probiotics (over placebo) on CRTI as compared to no probiotic consumption. The model was able to correctly reproduce the current incidence pattern of CRTI during a winter season, in France. The effect of using probiotics on CRTI duration or incidence as per the meta-analyses was then simulated. The estimated public health impact was significant for France, a country of 65 million inhabitants: up to 6.6 million fewer days with CRTI, 473.000 avoided prescriptions of antibiotics and 1.45 million sick leave days could be saved. The budget saving is commensurable: up to €254 million for the society, €131 million for the family and €37.7 million for the French NHS. It is important to interpret these results in a population-based framework, as the average benefit shown in the meta-analyses (e.g. 0.77 CRTI days averted) might seem minor at the individual level, while the effect applied to a broad population becomes significant. Importantly, the analysis was conducted on the CRTI patients consulting their general practitioner. In France this represents 37% of the population suffering from flu symptoms with fever and only 1% of those with common colds symptoms [47, 48]. Our analysis thus ignored non-prescribed absenteeism and presenteeism. This leads to an important underestimation of productivity losses related to CC, reported as high by several authors [12, 49].

In addition, the winter season studied (2011–2012) was associated with a low incidence rate of ILI (3,258 per 100,000 while rates since 2008–09 ranged from 4,385 to 6,344 per 100,000). The impact of probiotic consumption would be expected to be greater in seasons with higher incidence rate of CRTIs.

On the other hand, we remind that the modelization measured the maximum effect of using probiotics in the 3–79 years old population compared to no probiotic consumption. In reality, a proportion of the French population currently consumes probiotic products and will already experience the associated benefits, while generalized consumption is unlikely to be achieved. No reliable data on the actual consumption of probiotics in French families could be obtained, especially when it comes to differentiate occasional from regular users. This model however is designed to receive further data input on the actual consumption if this becomes available. A hypothetical value of one third (33%) of probiotics users in the general population aged 3–79 was tested. In this simulation, the number of averted CRTI days decreased from 2,4 million (base case YHEC scenario) to 1,6 million and the number of averted CRTI episodes decreased from 0.9 million (base case Cochrane scenario) to 0.6 million. Reduction of health care expenditures will decrease proportionally, but potential cost-savings remain of interest.

Another limitation of our analysis concerns the cost of the evaluated intervention (probiotics) that could not be precisely estimated given the range of available probiotic products. From the household and society perspectives the extra cost was estimated around 230€ (126–336€) for the CRTI season for an average family with 2 children, but could be lower if the probiotic replaces other traditional dairy products that are already part of the French traditional eating habits.

Compared to a classic pharmaco-economic analysis, in which the drug cost is generally set at the national or even European level, the cost of a food purchase does normally not weigh on the NHS expenses. In this regard, the impact of nutrition-associated health strategies on the NHS budget is comparable to the one of influenza vaccination policy: young healthy individuals not targeted by the national recommendations can decide to purchase the vaccine to avoid the flu, which leads to savings with no or limited extra expenses for the NHS. Simulations tools were successfully used in Europe to demonstrate the benefits of protecting/vaccinating healthy individuals against influenza from the NHS perspective [50–51]. Such tools may therefore be of interest in nutrition-oriented public health strategies as well.

Contrary to the above-mentioned tools, we did not include the indirect protection of individuals (called “herd immunity”, i.e. reduced disease’s opportunities to be passed on) arising from the averted CRTI cases/days in those using probiotics: modeling a dynamic transmission of CRTI would indeed require a lot more inputs and assumptions, especially on pathogens’ infectiousness, immunity duration and between-individuals contact patterns.

We focused on two determinist scenarios, and therefore sensitivity analyses are not reported in the results section. We estimated afterwards that the most impactful scenario (Cochrane) might avert between 500,000 and 1,000,000 CRTI cases per season, given the low and high values of the relative risk (RR = 0.65 to 0.81).

It should also be noted the inclusion of risk factors was assumed independent from each other. Most probably, the effect of having concomitant risk factors on the CRTI risk is less than the multiplication of the relative risks.

Further limitations include the extrapolation of the results to other countries and seasons: the model was applied to France. In terms of public health impact it is likely that a *pro rata* to the population size of other countries might give a rough estimate, especially for the neighboring countries because of free cross-border circulation in the European Union. Similar ILI incidence databases are however necessary for properly adapting the model inputs to another country. As said above, the 2011–2012 season used in the analysis was characterized by a low ILI incidence compared to average rate over the last five winter ILI epidemics.

Finally, we limited the effect of probiotics to environmental risk factors to mimic a consumer behavioral approach.

## Conclusion

The effect of probiotics on CRTI, as supported by two-meta-analyses and extrapolated with our model, is significant on public health and budget in a country like France and shows positive consequences to all economic agents. It benefits the NHS, the society, and the family. Children and people living in the community have higher incremental benefits, because of their higher exposure to respiratory viruses, in combination with an immune system that is either “under construction” (children) or “declining” (elderly) [52, 53]. Prevention is an important aspect of health. Hand washing and face masks have already showed some effectiveness in the control of spreading CRTI [54]. Probiotics could also be taken into consideration when searching for population-oriented strategies for limiting CRTI in primary health care and household settings during the winter season.

## Supporting Information

S1 Dataset Source values for building the TreeAge model. The values stored in this Excel file include all demographic information, risk factors prevalence, relative risks of CRTI, as well as source data on ILI + influenza incidence per week and age class. The values can be linked to a Treeage model.
(XLSX)
